# Differential Gene Expression from Midguts of Refractory and Susceptible Lines of the Mosquito, *Aedes aegypti*, Infected with Dengue-2 Virus

**DOI:** 10.1673/031.010.4101

**Published:** 2010-05-08

**Authors:** Olga L. Barón, Raul J. Ursic-Bedoya, Carl A. Lowenberger, Clara B. Ocampo

**Affiliations:** ^1^ Centro lnternacional de Entrenamiento e Investigaciones Médicas, Cali, Colombia; ^2^ Department of Biological Sciences, Simon Fraser University, Burnaby B.C., Canada

**Keywords:** suppressive subtractive hybridization

## Abstract

Suppressive subtractive hybridization was used to evaluate the differential expression of midgut genes of feral populations of *Aedes aegypti* (Diptera: Culicidae) from Colombia that are naturally refractory or susceptible to Dengue-2 virus infection. A total of 165 differentially expressed sequence tags (ESTs) were identified in the subtracted libraries. The analysis showed a higher number of differentially expressed genes in the susceptible *Ae. aegypti* individuals than the refractory mosquitoes. The functional annotation of ESTs revealed a broad response in the susceptible library that included immune molecules, metabolic molecules and transcription factors. In the refractory strain, there was the presence of a trypsin inhibitor gene, which could play a role in the infection. These results serve as a template for more detailed studies aiming to characterize the genetic components of refractoriness, which in turn can be used to devise new approaches to combat transmission of dengue fever.

## Introduction

Mosquitoes are the vectors of pathogens that cause important human diseases including malaria, filariasis, dengue, yellow fever among others (Paul et al. 2006). *Aedes aegypti* (Diptera: Culicidae) is the major vector of dengue viruses that cause more human mortality and morbidity than any other arthropod-transmitted viral disease (Alphey et al. 2002). An estimated 50 to 100 million cases of dengue fever occur annually, and 2.5 billion people are at risk of infection (Black et al. 2002; Guzman and Kouri 2002; Gubler 2004; Guha-Sapir and Schimmer 2005). There is no vaccine available, and there are no drugs to cure dengue fever. Dengue control is based on surveillance measures and mosquito control using insecticides and larval habitat reduction strategies (Spiegel et al. 2005). However, despite these efforts, the number of cases of dengue fever and dengue hemorrhagic fever continue to rise each year, and, therefore, alternative control avenues are being investigated.

Some of these efforts have focused on the genetic manipulation of insect vectors (Beaty 2000; Aksoy et al. 2001; Alphey et al. 2002) to modulate characteristics such as vector competence, the intrinsic ability of a vector to transmit a pathogen (Woodring et al. 1996). Recent advances in molecular biology and the availability of genomic databases have enabled the development of new strategies for the control of vector-borne diseases. Manipulation of vector competence requires extensive knowledge on the molecular aspects of vector-parasite interactions. In this context, transgenic techniques have been used to introduce and achieve expression of foreign, antipathogenic genes in insect vectors (Aksoy et al. 2001; Dotson et al. 2003; Abraham et al. 2005; Riehle and Jacobs-Lorena 2005). In *Ae. aegypti*, an engineered construct of Sindbis virus has been used to express insect immune peptides (Cheng et al. 2001) or heterologous virus sequences to induce an RNA interference-like response to the target virus (Adelman et al. 2001).

Population genetics studies of vector competence in *Ae. aegypti* have demonstrated a high variation of this characteristic among different populations (Tabachnick 1982; Apostol et al. 1996; Paupy et al. 2000; Vazeille-Falcoz et al. 2001; Garcia-Franco et al. 2002; Gorrochotegui-Escalante et al. 2002). Susceptible and refractory strains obtained using isofemale selection demonstrated an association of vector competence with genetic components that can be affected by environmental changes (Wallis et al. 1985; Miller and Mitchell 1991). Quantitative genetic studies have revealed that at least two genes or sets of genes control vector competence (Bosio et al. 2000; Black et al. 2002). These studies have allowed associating a genetic component with vector competence, but the role of specific receptors or immune response-related genes that modulate arbovirus infection and replication, and the factors that determine resistance or susceptibility to arboviruses such as dengue, are still unknown.

Insects have developed precise mechanisms to protect themselves against bacterial, fungal, and parasitic infections. This immune response is innate and, depending on the type and size of the pathogen, may involve a combination of phagocytosis (Pearson et al. 1995; Kocks et al. 2005; Watson et al. 2005; Lemaitre and Hoffmann 2007), encapsulation and melanization (Karlsson et al. 2004; Bidla et al. 2005; Paskewitz et al. 2006), and production of lethal antimicrobial peptides (Lowenberger 2001; Imler and Bulet 2005). Immune reactions begin with the recognition of cell-surface molecules of pathogens, known as pathogen-associated molecular patterns, by specific insect receptors (pattern recognition receptors) (Michel and Kafatos 2005). This interaction between pathogen-associated molecular patterns and pattern recognition receptors selectively activates either of two intracellular signaling pathways. In *Aedes*, as in *Drosophila*, Gram + bacterial and fungal infections induce the Toll pathway, which results in the translocation of the NF-KB transcription factor, and Gram — bacteria triggers the Imd pathway, which results in the nuclear translocation of Relish (a NF-κB-type transcription factor) and the induction of antimicrobial peptides such as cecropin and defensin (Lowenberger 2001; Bartholomay et al. 2004). Although these immune pathways are conserved among mosquito species, there are differences in the molecules involved (Hoffmann and Reichhart 2002; Shin et al. 2003; Meister et al. 2005). Despite the knowledge of antimicrobial responses, the immune response in mosquitoes against viruses such as dengue has not been thoroughly defined (Sanders et al. 2005). Other insect-virus models such as *Drosophila*/Drosophila virus C suggest that, in addition to Toll and Imd, a third pathway, Jak/Stat, functions as a part of an antiviral innate immune response (Dostert et al. 2005; Zambon et al. 2005). In vertebrates, apoptosis is the first response to viral infections and later stimulates the adaptive immune system. This mechanism has also been described in some insect models infected with baculovirus as an antiviral defense strategy, but the role of apoptosis as an immune response has not been well-characterized in insects that transmit human parasites (Clarke and Clem 2003; Cooper et al. 2007a), despite the fact that apoptotic-like activity has been associated with *Plasmodium* infection in *Anopheles gambiae* (Al-Olayan et al. 2002). Recent studies in *Ae. aegypti* have identified and characterized two initiator caspases associated with apoptosis pathways, suggesting that this immune response might function as one of the mechanisms that insect vectors use to regulate the establishment and replication of intracellular parasites such as viruses (Cooper et al. 2007a, 2007b).

Although *Ae. aegypti* is the main vector of dengue virus, there are populations or fractions of populations that do not permit virus development, presumably because they have biological barriers that impair the establishment and dissemination process (Black et al. 2002). A high variability in vector competence among local populations of *Ae. aegypti* in Cali, Colombia ranging between 19% to 60% was identified (Ocampo and Wesson 2004), indicating the presence of naturally susceptible and refractory mosquitoes to Dengue-2 virus with different infection barriers. The midgut infection barrier is one of the initial mechanisms that viruses must overcome to establish a successful infection and is one that might be genetically altered to render mosquitoes resistant to arboviruses. Therefore, it is the focus of this study.

The differential expression of midgut genes between susceptible and refractory *Ae. aegypti* after exposure to Dengue-2 virus is reported here. The functional annotation of pathogen-specific vector-expressed sequence tags (ESTs) that could play a role in determining or contributing to vector competence in *Ae. aegypti* are also described.

## Materials and Methods

### Study rationale

In an attempt to identify factors that determine the susceptibility of *Ae. aegypti* to dengue virus, midgut gene expression was evaluated in *Ae. aegypti* individuals that were susceptible or refractory to Dengue-2 virus 48 h post infection.

### Mosquito strains

*Ae. aegypti* were collected in different localities from the city of Cali, Colombia and colonized at the Centro Internacional de Entrenamiento e Investigaciones Médicas insectary at 26 ± 2° C with 80% relative humidity and a 12:12 lightdark photoperiod. *Ae. aegypti* (Rockefeller strain) provided by the Centers for Disease Control and Prevention (Puerto Rico) were maintained in the insectary and were used as positive and negative controls in all PCR assays. The variability of susceptibility of different local populations of mosquitoes to Dengue-2 was described previously (Ocampo and Wesson 2004), indicating the presence of susceptible and refractory mosquitoes in Cali. To increase the number of refractory mosquitoes, an isofemale selection was carried out. For this selection, females were allowed to feed on an infectious blood meal and were placed individually in oviposition cages. After 14 days of incubation, the phenotype of the mother, with respect to the biological barriers, was identified (midgut infection barrier, midgut escape barrier, and susceptibility) as described by Bennett et al. (2005). The eggs of each female were collected. These eggs were hatched and the emerging adults were sorted based on the phenotype of the mother. These descendents from susceptible and midgut infection barrier females were infected with a Dengue-2 infectious blood meal and midgut tissues were collected 48 h later.

### Virus maintenance and mosquito infection

Dengue-2 virus New Guinea C strain, freshly grown in C6/36HT (*Aedes albopictus* larvae cells) was used in oral challenges. Infected cells were incubated for 14 days at 32° C in L 15 medium supplemented with 2% heatinactivated fetal bovine serum, 1% penicillin/streptomycin, and 1% L-glutamine (Higgs and Beaty 1996). Virus and cells were harvested and collected in a 15-ml conical centrifuge tube. Aliquots of the infected cell suspension and the mixture of blood and virus before and after the infection process were titred using the methodology described by Bennett et al. (2002). Titres in the cell suspensions ranged from 10^8^ to 10^8.5^ TCID50/ml in all oral challenges. Oral infections were done in a BSL2+ (biosafety laboratory) insectary with eight protection barriers. Artificial blood feeding was carried out using a membrane feeder. Infected blood was prepared by mixing defibrinated rabbit blood and Dengue-2 virus suspension (1:1 v/v) (Higgs and Beaty 1996). Adult females, six to seven days after eclosion, were deprived of sucrose and water for 24 h prior to blood feeding. Mosquitoes were allowed 1 to 1.5 hours to feed *ad libitum*. Fully engorged mosquitoes were separated and kept in a separate cage with access to a 10% sugar solution.

### Tissue dissection and RNA isolation

Forty-eight hours after infection, midguts from bloodied mosquitoes were dissected on a chilled table and thoroughly rinsed in cold DEPC-PBS to remove traces of the blood meal. Tissues were stored individually in RNA later (Qiagen, www.qiagen.com) at -20° C for subsequent RNA isolation. The RNA later solution was removed by pipetting. Total RNA extraction from individual midguts was performed using RNeasy Mini Kit (Qiagen) according to the manufacturer's instructions. Total RNA was quantified using a NanoDrop Spectrophotometer ND-1000 (NanoDrop Technologies, www.nanodrop.com).

### Detection of infection

An established nested reverse transcriptase PCR protocol (Lanciotti et al. 1992) was standardized using three groups of *Ae. aegypti* Rockefeller strain to determine the sensitivity of reverse transcriptase PCR to detect the virus in individual midguts. These groups were: mosquitoes inoculated with Dengue-2 virus (positive controls), infected-bloodfed mosquitoes, and non-bloodfed (naïve) mosquitoes. Midguts were dissected, and RNA was extracted as described above.

In the reverse transcriptase PCR reactions, 50 ng of total RNA were reverse transcribed in a 20 µl reaction mixture containing 1X firststrand buffer (50 m*M* Tris-HCl (pH 8.3), 75 m*M* KCl, 3 m*M* MgCl_2_), 5 m*M* DTT, 500 µ*M* of dNTPs mix, 50 pmol of primer D2 (5′TTGCACCAACAGTCAATGTCTTCAGGT TC-3′) and 50 units of Superscript II Reverse Transcriptase (Invitrogen, www.invitrogen.com). Reverse transcription was conducted at 42° C for 60 min and 95° C for 5 min. The resulting cDNA was used in a 50 ìl PCR reaction containing 1X PCR buffer (50 m*M* KCl, 10 m*M* Tris-HCl (pH 9.0), 0.1% Triton® X-100), 1.5 m*M* MgCl_2_, 125 µ*M* of each dNTP, 50 pmol of primers D1 (5′TCAATATGCTGAAACGCGCGAGAAACC G-3′) and D2, and 0.05 U of Taq DNA polymerase (Invitrogen). PCR was performed with the following parameters: 95° C for 1 min; 30 cycles of 94° C for 45 s, 58° C for 45 s, and 72° C for 1 min; and a final extension at 72° C for 7 min. A second-round PCR was run with a 1:100 dilution from the first PCR reaction. PCR was performed under the same conditions used for the primary PCR with the following modifications: primer D2 was replaced with the Dengue-2 virus-specific primer TS2 (5′-CGCCACAAGGGCCATGA ACAG-3′, 50 pmol) and 35 amplification cycles were used. PCR products were resolved by 2% agarose gel electrophoresis with a 100bp DNA ladder (Invitrogen) stained with ethidium bromide and visualized under UV light.

### Subtractive library construction

According to the PCR result, positive (infected) and negative (non-infected) midgut RNA samples for each phenotype were pooled separately. A total of 60 midguts were pooled for each phenotype to obtain sufficient RNA to generate the suppressive subtractive hybridization (SSH) libraries. All RNA pools were precipitated and treated with DNAse (Qiagen).

Total RNA from each pool was used to generate cDNA using the SMART PCR cDNA Synthesis Kit (Clontech, www.clontech.com) according to the manufacturer's recommendations. This procedure generated a sufficient quantity of high-quality cDNA from small quantities of RNA for subtractive library procedures.

Libraries were built using PCR-Select cDNA Subtraction kit (Clontech) according to the manufacturer's specifications. SSH is a PCR-based technique that facilitates the detection of differentially expressed sequences in two samples by allowing exponential amplification of differentially expressed genes and suppressing the amplification of sequences common to both samples. This technique has been used previously to identify differentially expressed genes in *Rhodnius prolixus* in response to pathogens and parasites (Ursic-Bedoya and Lowenberger 2007).

Three subtractive libraries were constructed: a library of differentially expressed genes in mosquito midguts after injection of *Escherichia coli* (control library), and two cDNA libraries from the midguts of Dengue-2 virus-susceptible and virus-refractory mosquitoes after the ingestion of a Dengues-infected blood meal. A predictable *E. coli* control library was built to confirm that the small amount of RNA available for the dengue-susceptible and dengue-refractory was sufficient to build SSH libraries.

The products of the subtracted procedure were ligated into pGemT Easy plasmid vector (Promega, www.promega.com) and transformed by heat shock into *E. coli* JM109 ultra-competent cells (Promega) as previously described by Ursic-Bedoya and Lowenberger (2007). Putative transformant colonies were grown overnight in 96-well plates with 100 µl of LB medium and 0.1 µl of ampicillin (100 µg/µl). For forward and reverse libraries, a total of 384 colonies (four plates) were selected from each library for differential screening. For the bacteria-induced library, 192 colonies (2 plates) were selected.

### Subtraction efficiency analysis and differential screening

The subtraction efficiency of the SSH process in all libraries was measured using PCR to amplify, before and after subtraction, a housekeeping gene that should be present in both libraries and an induced gene that should be present in only the enriched library. The Beta-actin sequence from *Ae. aegypti* with the primers actinF637LVP: 5′- ATTAAGGAGAAGCTGTGCTACGTC and actinR942LVP: 5′-CATACGATCAGCA TTACCTGGG was used. The PCR program was 94° C for 1 min, followed by 33 cycles of 94° C for 20 s, 60° C for 20 s, 68° C for 30 s and a final extension of 68° C for 2 min. To measure a differentially expressed gene, the *Ae. aegypti* lysozyme was used as described by Ursic-Bedoya and Lowenberger (2007).

Both midgut subtracted libraries were screened for differentially expressed ESTs using the PCR-select differential screening kit (Clontech) following the manufacturer's instructions. One hundred and fifty nanograms from the forward and reverse subtracted libraries were used to create a ^32^P-labeled probe by random priming. Forward and reverse subtracted probes were hybridized in individual tubes with Hybond+ DNA membranes (Amersham Biosciences, www.gelifesciences.com) containing individually spotted EST clones (Ursic-Bedoya and Lowenberger 2007). ^32^P-labeled probes and target EST membranes were hybridized at 65° C for 2.5 h in a rotatory oven using Rapid—Hyb buffer (Amersham Biosciences). Following hybridization, the membranes were washed with low stringency (2X SSC, 0.5% SDS; 3 times, 20 min each) and high stringency (0.2X SSC, 0.5% SDS; 3 times, 20 min each) buffers at 65° C to eliminate non-specific binding due to excess probe. Membranes were exposed to a Kodak BioMax MS film (Eastman Kodak, www.kodak.com) overnight at room temperature. Selected colonies (strong signal with the forward and low signal with the reverse subtracted probe) were sent to BC Genome Sciences Centre (Vancouver, BC) for plasmid purification and sequencing.

### Sequence analysis

Sequence homology searches were carried out using NCBI's BLAST-X (http://www.ncbi.nlm.nih.gov/blast/) against nr databases with default parameters. The best annotated matches were retained. Sequences with no significant matches in NCBI's databases were translated in all possible reading frames and were analyzed using INTERPRO SCAN to identify conserved protein domains so that putative function could be assigned. Additionally, sequences with no significant match in the NCBI program were analyzed against the VECTORBASE database (www.vectorbase.org/Tools/BLAST). Homologies were considered statistically significant if they generated an Expect value (E) < 0.1. The EST sequences reported in this paper were submitted to the NCBI dbEST and assigned accession numbers 56768811 to 567689975 (GenBank FG107129 to FG107293).

## Results

### Library of ESTs found only in Dengue-2 susceptible midguts

This library was created using susceptible insects as the tester and refractory insects as the driver (forward library) in order to identify genes differentially expressed in the susceptible population. In order to increase selection of susceptibility-related genes, recombinant colonies were differentially screened by hybridization with forward (susceptible) and reverse (refractory) probes. Differentially expressed and over-expressed clones were selected. Of the 384 clones screened, only 125 were confirmed to be up-regulated by differential screening as described above.

All 125 clones were sequenced, from which 22 clones (17.6%) did not have similarities with other sequences in the databases (data not shown). A similarity search identified 57 putative genes from 103 clones that matched with annotated sequences in databases (Tables 1, 2). All identified genes were clustered in functional groups according to their putative function as cytoskeleton, nucleic acid binding, metabolism, transcription factors, immunity, ion binding and transport, receptors, mitochondrial, signaling and digestion genes. Out of 57 ESTs, 9 sequences coded for ribosomal genes (normally repressed in the suppressive subtractive hybridization), and 15 clones corresponded to hypothetical proteins. Only 11 of the putative genes had more than one copy, and 4 of them were highly repetitive (more than 3 copies) as DNA binding, Cytoskeleton, Cytochrome P450 and calcium ion binding genes that are potentially related with intracellular infections, cellular distress, and immune responses.

### Library of ESTs found only in Dengue-2 refractory midguts

A total of 384 clones were spotted on membranes, but only 40 were confirmed to be up-regulated in the refractory tissues compared with the susceptible library after hybridization with the forward and reverse probes. Of those 40 clones, 5 had no significant match to other genes in the databases (data not shown). Bioinformatic analyses showed 23 (65.7%) of the EST sequences corresponded to different putative genes (Tables 3, 4). Among these transcripts, 9 sequences were hypothetical proteins. Four clones (1%) had more than one copy, but, in contrast with the susceptible library, they were not highly repetitive. Subtraction efficiency analysis by PCR showed better quality in this subtraction than in the susceptible library since only one ribosomal gene was detected.

**Figure 1.**
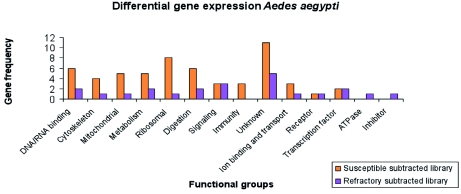
Differential gene expression in susceptible and refractory subtracted libraries. High quality figures are available online.

Contrary to the susceptible subtracted library, genes related with cellular stress or immune responses were not detected (Figure 1). An interesting finding was the presence of a trypsin inhibitor gene that was differentially expressed in this library (Table 3). This protein could affect dengue virus infection; the inhibition or knockdown of specific trypsin molecules has been reported to reduce (Molina-Cruz et al. 2005) or increase (Brackney et al. 2008) the infectivity of dengue virus in *Ae. aegypti*.

**Table 1.**
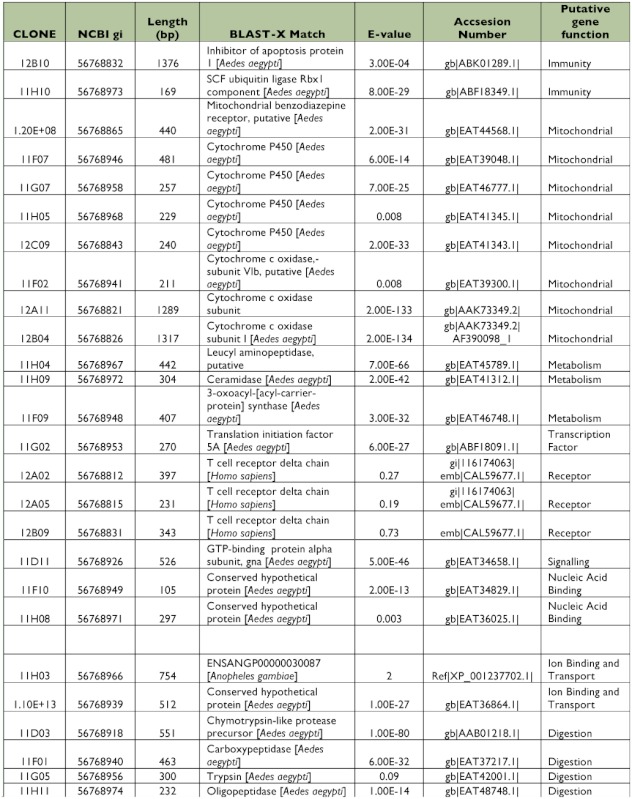
EST identified in the susceptible midgut subtracted library using BLAST database.

**continued:**
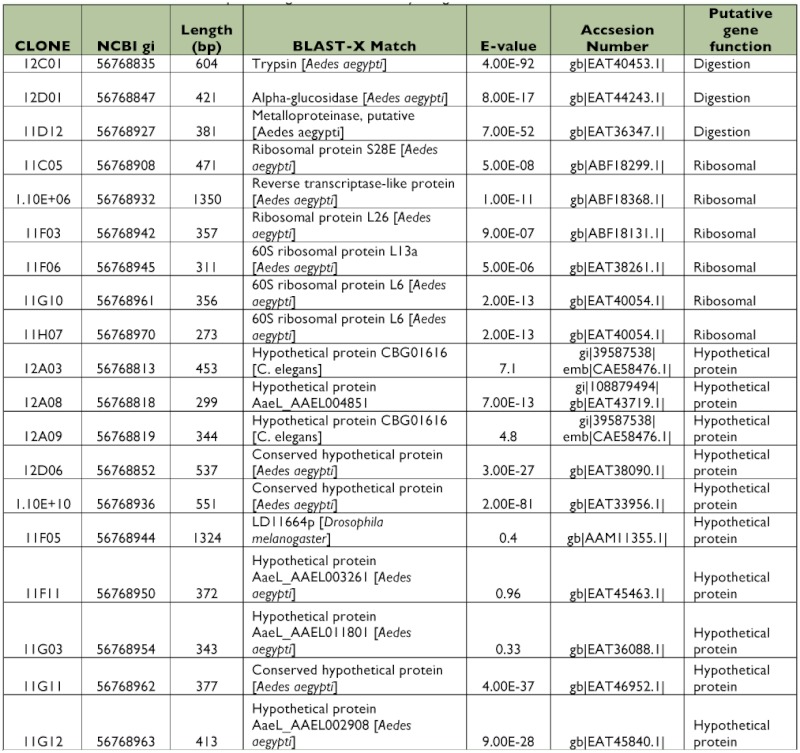

**Table 2.**
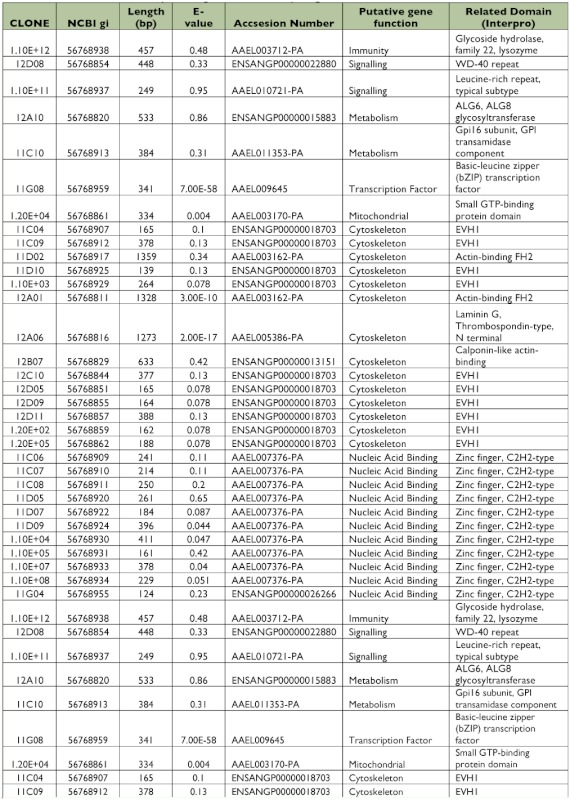
EST identified in the susceptible midgut subtracted library using VECTORBASE database.

**continued:**
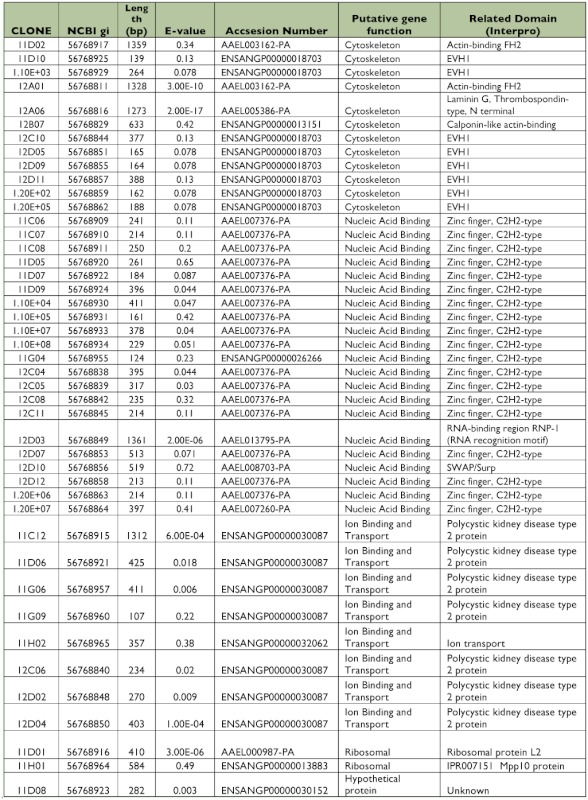

**continued:**
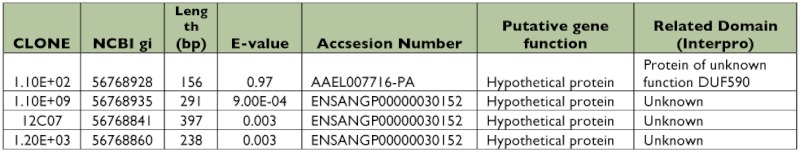

## Discussion

Insect innate immune responses have been studied against bacteria, parasites, and fungi, but antiviral responses have not been well-characterized (Sanders et al. 2005). In eukaryotic organisms, antiviral innate immune mechanisms involve mechanical barriers (Paskewitz and Christensen 1996; Schmid-Hempel 2001; Schmid-Hempel 2005), gene silencing (RNAi and miRNA) (Ausubel 2005; Fritz et al. 2006; Wang et al. 2006), production of humoral and effector mechanisms (Cherry and Silverman 2006; Seth et al. 2006; Zhong et al. 2006) and apoptosis (Everett and McFadden 1999; Irusta et al. 2003).

It is known that the mosquito immune response mechanisms are similar to those of *Drosophila melanogaster*. However, *D. melanogaster* is not a vector of arboviruses, and therefore, certain immune mechanisms could be specific to mosquitoes such as *Ae. aegypti*. Although there are conserved genes among *D. melanogaster, An. gambiae* and *Ae. aegypti*, immune genes are the most divergent group even among closely related species. An estimated 285 genes related to immune response, apoptosis and oxidative stress were identified in *D. melanogaster*, while 338 such genes were identified in *An. gambiae*, and *353* in *Ae. aegypti* (Nene et al. 2007). Phylogenetic studies indicate that genes related to pathogen recognition and signaling intracellular pathways are conserved in the three species. Genes encoding effector molecules such as antimicrobial peptides, however, may be more diverse or species specific (Nene et al. 2007; Waterhouse et al. 2007).

**Table 3.**
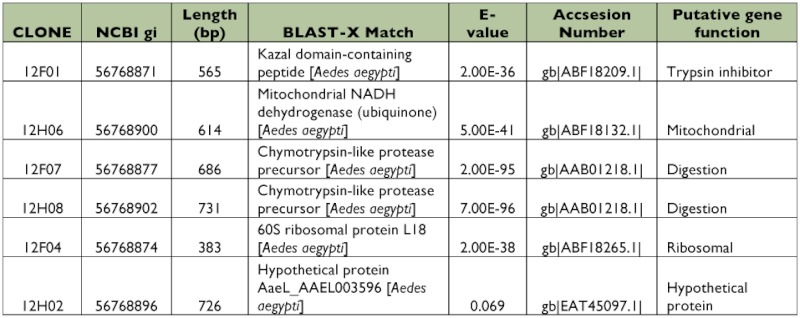
EST identified in the refractory midgut subtracted library using BLAST database.

**Table 4.**
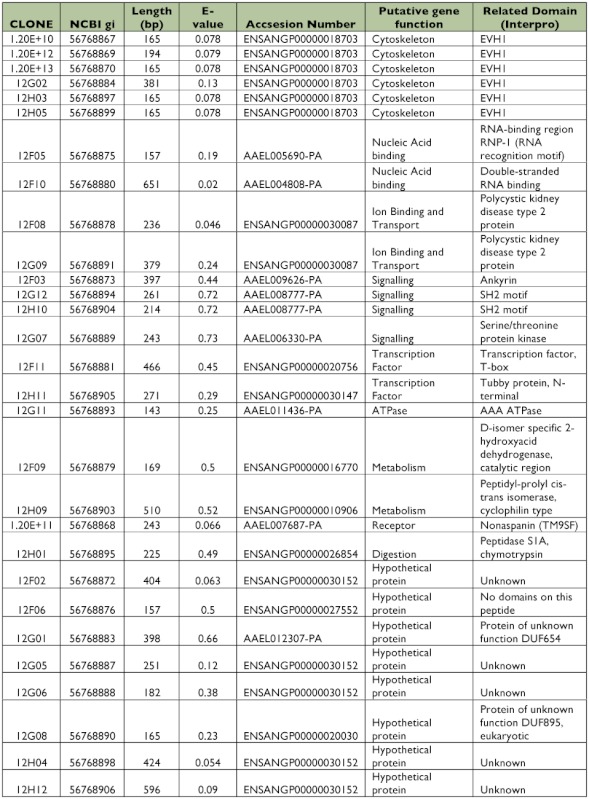
EST identified in the refractory midgut subtracted library using VECTORBASE database.

Viral infection studies in *D. melanogaster* have revealed the role of RNA interference, Toll, Imd, Jak-Stat pathways and apoptosis mechanisms as antiviral responses. The diverse kinds of responses seen in these experiments depend on the virus life cycle and the method used in the experimental infections (oral or intrathoracic inoculation) (Keene et al. 2004; Lemaitre and Hoffmann 2007). This study focused on identifying immune response related genes in the mosquito midgut, the primary barrier that the virus must overcome in order to infect the vector. It has been estimated that only 40–50% of the initial viral load is capable of crossing the midgut barrier (Paskewitz and Christensen 1996).

In this study, differential expression analysis showed that susceptible *Ae. aegypti* express a higher number of metabolic and immune response-related genes than the refractory mosquitoes. Immune genes were primarily associated with both inhibition and execution mechanisms of apoptosis. The identified molecules associated with this process were: inhibitor of apoptosis, ubitiquin ligase complex, Cytochrome c, cytoskeleton genes and proteins with calcium ion binding domain. The over-expression of these molecules suggests that cell stress and apoptosis could be playing a key role during the infection process. Other studies have hypothesized that mosquitoes might use apoptosis to eliminate intracellular parasites such as viruses (Cooper et al. 2007a, 2007b), and that viruses might prevent it by activating the expression of inhibitors of apoptosis. The presence of anti and pro-apoptotic molecules suggests that the virus manipulates the cellular machinery to allow its replication and dissemination, as has been demonstrated in cell cultures (O'Brien 1998). To this point, the differential apoptotic responses in refractory and susceptible *Ae. aegypti* strains after ingesting Dengue-2 were not measured, but experiments are ongoing.

In contrast, an upregulation of immune-related genes was not observed in refractory mosquitoes possibly because: a) the virus does not enter the midgut cells, b) the virus cannot establish the infection, c) there are early events of apoptosis that eliminate virus-infected cells, d) the technique used or availability of all genes in the databases did not allow the identification of other potential refractory mechanisms, or e) the mechanism is not mediated at the transcriptional level, but may be mediated by previously produced zymogens. In this context, an interesting finding in the refractory strain was the presence of a trypsin inhibitor gene. Trypsin is a digestive enzyme that has been implicated in the dengue virus infectious process (Molina-Cruz et al. 2005, Brackney et al. 2008). It is possible the trypsin inhibitor gene found in this library could affect dengue infection in the mosquito. If it is supported, this finding may suggest that refractoriness may not be due to an active immune response based on well-known and well-characterized immune processes (i.e. antimicrobial peptide expression, phagocytosis activation by TEP, phenoloxidase, melanization) but may be a function of the virus not being able to exit midgut epithelial cells, if it was able to enter initially.

The SSH technique and its ability to identify differentially expressed genes in the midguts of Dengue-susceptible and -refractory individuals were validated, but there are few similar studies with which to compare the results. The *E. coli*-injected control library identified some immune peptides that have been demonstrated broadly in other studies as cecropin, serine proteases, and conserved protein related to cell death (data not shown).

The limitations of the SSH technique were recognized in terms of its sensitivity, since it detects only highly over-expressed genes; however, this technique was selected for its accessibility and as a primary step in identifying potential differences between the susceptible and refractory strains. Additionally, this technique allowed us to work with the small amount of RNA that was obtained by pooling individual midguts that were previously tested.

As described above, many of the ESTs generated in this study have no known match in the databases, and they will continue to be submitted to the growing number of databases as more motifs and genomes are sequenced. The lack of match may indicate a true lack of comparable sequences in the databases, or may indicate that the ESTs map to 3′ and 5′ untranslated regions. The ESTs were examined, but none of the classic motifs found in 3′ untranslated regions were found.

Some of the more interesting proteins to which the ESTs map, and which could play a key role in the susceptibility or refractoriness to Dengue-2 virus in *Ae. aegypti*, are discussed below. Further studies are underway to evaluate these molecules in more detail.

## Inhibitor of apoptosis

Insect inhibitor-of-apoptosis proteins contain two baculoviral inhibitor-of-apoptosis repeat domains and a Zinc RING domain. Inhibitors of apoptosis impede activation of initiator and executioner caspases preventing either their dimerization or their binding to the active catalytic site of these enzymes (Huh et al. 2007; Leu et al. 2007). Some inhibitors of apoptosis have been identified and characterized in insects, but their significance during arbovirus infection in mosquitoes has not been completely elucidated (Blitvich et al. 2002; Li et al. 2007; Bryant et al. 2008). In an *An. gambiae* functional genomics study, 6 inhibitors of apoptosis were differentially expressed during *Plasmodium berghei* infection in midgut epithelial cells (Vlachou et al. 2005). Likewise, there was an upregulation of apoptosis related-molecules in *Ae. aegypti* infected with Sindbis virus, and, among these, one inhibitor of apoptosis was over-expressed (Sanders et al. 2005). What is most interesting in this study is, for the first time, the identification of apoptosis as an antiviral response in a natural Dengue/*Ae. aegypti* model using wild mosquito populations.

Apoptosis as an immune response mechanism in vertebrates has been widely described. However, in invertebrates, this process has not been clearly characterized. The results of this study are consistent with other studies, suggesting this cell death process is one of the mechanisms that insect vectors use to regulate intracellular parasites such as viruses (Cooper et al. 2007a, 2007b).

## Ubiquitin ligase complex

This enzyme complex participates in protein degradation by the proteasome in a number of key biological processes, including cell cycle progression and signal transduction (Maniatis 1999). Ubiquitin-dependent proteolysis controls the abundance of many regulatory proteins and caspase activation (Wojcik 2002; Arama et al. 2007). Several studies have shown a crosstalk between the apoptotic pathways and the ubiquitin- proteasome system (Orlowski 1999; Schreader et al. 2003; Arama et al. 2007). During the cell death process, the ubiquitin ligase complex promotes caspase activation via ubiquitination and degradation of caspase inhibitors. Inhibitors of apoptosis, with ubiquitin protease ligase (E3) activity in their RING finger domain, undergo auto-ubiquitination and degradation by proteasome (Grimm and Osborne 1999; Hu and Yang 2003). The ubiquitin-mediated pathway also regulates NF-KB factors in activation. In the *D. melanogaster* Toll pathway, upregulation of ubiquitin ligase levels leads to the degradation of Cactus, allowing the nuclear translocation of Dorsal (Spencer et al. 1999).

## Cytochromes (P450 and *c*)

Cytochromes are proteins involved in several cellular functions such as oxidative stress, respiration, apoptosis and xenobiotic metabolism (Scott and Kasai 2004; Arama et al. 2006). In mammal cells, release of Cytochrome c and other proapoptotic molecules induce caspase activation and cell death via the mitochondrial apoptosis pathway (Hengartner 2000; Wang 2001). In insects, the role of mitochondria and Cytochrome c in apoptosis has been contradictory and not completely characterized (Abdelwahid et al. 2007; Goyal et al. 2007). Some reports have suggested that Cytochrome c release is not a necessary step to trigger apoptosis in some *D. melanogaster* cells (Dorstyn et al. 2004; Abdelwahid et al. 2007). In contrast, Liu (2007) showed that Cytochrome c is required for caspase activation in *Baculovirus*-induced apoptosis in *Spodoptera litura* cells.

## Trypsin inhibitor

Trypsin, a major midgut proteolytic enzyme, is essential for blood digestion in *Ae. aegypti* (Noriega and Wells 1999) and its presence has been reported to enhance arbovirus infectivity, replication, and dissemination (Ludwig et al. 1991; Xu et al. 1997, Molina-Cruz et al. 2005). The addition of soybean trypsin inhibitor has been reported either to increase midgut infection rates (Brackney et al. 2008) or to decrease Dengue-2 infectivity and dissemination (Molina-Cruz et al. 2005).

Feeding different trypsin inhibitors blocked early trypsin activity but did not reduce late trypsin expression, and RNAi knockdown of early trypsin did not affect late trypsin expression (Lu et al. 2006). The interaction between the expression of early and late trypsin is not clear. RNAi silencing of chymotrypsin, early and late trypsin had no effect on Dengue-2 infectivity whereas RNAi knockdown of a third trypsin, 5G1, reduced trypsin activity and increased dengue infectivity in the midgut (Brackney et al. 2008). These studies suggest that some midgut serine proteases, acting through digestion or direct activity on viral proteins, may affect Dengue-2 infectivity of *Ae. aegypti*. The presence of the EST with high homology to a trypsin inhibitor (Table 3) in the refractory strain suggests that inhibition of trypsin activity as a digestive enzyme or in cleaving viral proteins could contribute to the refractoriness of the wild *Ae. aegypti* population used in this study. However, further studies are required to determine which trypsins are affected by this inhibitor and subsequently their specific roles in limiting or enhancing Dengue-2 infectivity.

The data presented here have identified differences in gene expression between feral populations of *Ae. aegypti* that are naturally susceptible or refractory to Dengue-2 virus. There was an over-expression of numerous molecules and the involvement of diverse biological processes showed the complexity of viral infection and immune responses against the virus. The functional characterizations of the apoptosis-related genes have begun to be evaluated in order to elucidate their role in the susceptible or refractory phenotypes. In addition, more investigations need to be done in order to evaluate whether known immune pathways (Toll and Imd) are activated after dengue virus infection.

## Abbreviations

EST: expressed sequence tag;
SSH: suppressive subtractive hybridization
